# Type III endoleaks following endovascular aortic repair: a case series

**DOI:** 10.1093/jscr/rjag887

**Published:** 2026-10-04

**Authors:** Buraq Ahmed, Oliver Chan, Maite Del Campo Martinez, Hariesha Pathmaraj, Chris Grieco

**Affiliations:** School of Clinical Medicine, University of Cambridge, Addenbrooke's Hospital, Hills Rd, Cambridge CB2 0SP, United Kingdom; School of Clinical Medicine, University of Cambridge, Addenbrooke's Hospital, Hills Rd, Cambridge CB2 0SP, United Kingdom; School of the Biological Sciences, University of Cambridge, 17 Mill Lane Cambridge CB2 1RX, United Kingdom; School of Clinical Medicine, University of Cambridge, Addenbrooke's Hospital, Hills Rd, Cambridge CB2 0SP, United Kingdom; Radiology Department, Cambridge University Hospitals NHS Foundation Trust, Addenbrooke's Hospital, Hills Rd, Cambridge CB2 0SP, United Kingdom

**Keywords:** type III endoleak, EVAR, TEVAR, aneurysm, thoracic aorta, interventional radiology

## Abstract

Type III endoleaks, from disconnection between modular stent-graft components (type IIIa) or fabric defects within a limb (type IIIb), are uncommon but potentially life-threatening, transmitting systemic pressure into the aneurysm sac and carrying a high rupture risk. We describe three men with late type III endoleak failure after complex thoracic aortic repair. One presented with rupture at a mid-thoracic modular overlap, salvaged by emergency relining via a hostile femoral cut-down. A second, under surveillance for an asymptomatic endoleak managed conservatively given a static sac, later ruptured at a separate site and died despite emergency relining. A third developed a type IIIb fabric failure in a highly angulated aortic arch, salvaged using a conformable stent-graft and left-ventricular through-and-through wire support. These cases show type III endoleaks may be radiologically subtle yet rapidly fatal, sac stability alone should not determine management, and complex arch morphology demands tailored device selection and access planning.

## Introduction

Endovascular aneurysm repair (EVAR) and thoracic endovascular aortic repair (TEVAR) have changed the landscape of thoracic and abdominal aneurysmal and dissecting disease management since their introduction. Whilst they surpass open repair with regards to inpatient or 30-day mortality rates and discharge time, they show increased rates of secondary intervention, especially due to endoleaks [1]. With an incidence of 2%–4.5% [2], type III endoleaks occur at junctions of stent-graft components (type IIIa) or fabric defects (type IIIb). Though most type III endoleaks occur due to modular separation (85%) [3] rather than fabric tears (15%) [3], they both transmit full systolic pressure into the aneurysmal sac, increasing risk of rupture and death [4].

Current Society for Vascular Surgery (SVS) and European Society for Vascular Surgery (ESVS) guidelines for both abdominal and thoracic aortic repair regard type I and type III endoleaks as technical failure of endovascular repair and recommend prompt intervention irrespective of aneurysm sac size, because persistent pressurization confers a high rupture risk [5–8].

This report describes three illustrative cases of type III endoleak after thoracic and thoracoabdominal endovascular repair, emphasizing differing mechanisms of late failure, technical approaches to relining, and the dilemma posed by patients in whom elective re-intervention is judged too hazardous. Written informed consent was obtained from the patients for publication of this case report and any accompanying images.

## Case series

### Case 1 – Ruptured type III endoleak in the mid-descending thoracic aorta

A 73-year-old man presented to a district general hospital with acute chest pain and hypotension. He had chronic kidney disease and a prior open aortobifemoral bypass. Approximately 7 months earlier (late 2023) he had undergone TEVAR at another centre with a modular thoracic stent-graft for a descending thoracic aneurysm, complicated by bilateral femoral seromas and pseudo-aneurysms. The index device, the surveillance protocol in place and any interval imaging were not available in the transferred records, so whether the endoleak was present on earlier surveillance is unknown. Computed tomography (CT) angiography demonstrated active contrast extravasation from the mid-descending thoracic aorta at an overlap zone between stent-graft modules, with a large left haemothorax, consistent with rupture from a type III endoleak (Fig. 1a and b). As this exceeded local endovascular capability, he was rapidly transferred to the regional tertiary centre.

**Figure 1:**
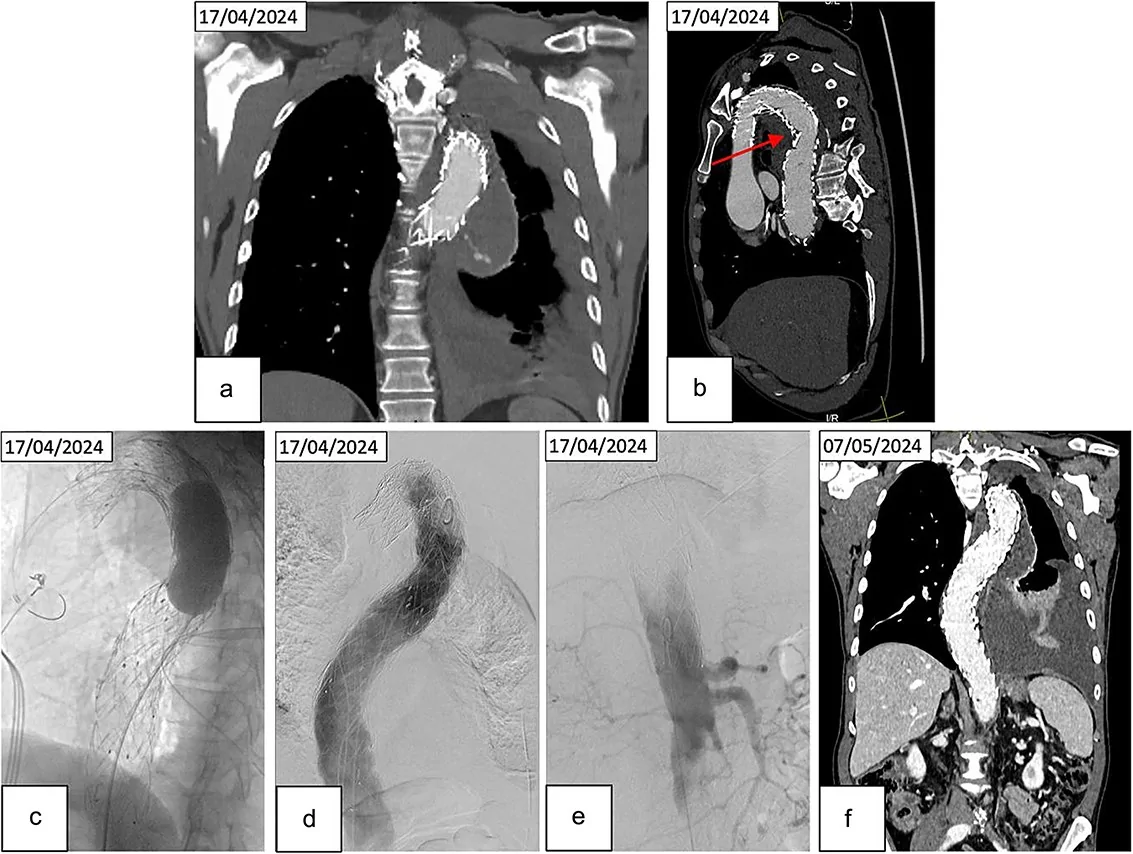
*Case 1: Ruptured mid-thoracic type III endoleak and thoracic relining (panels a–f).* (a) Pre-transfer CT angiography from the referring hospital showing contrast extravasation from the mid thoracic aorta with large left haemothorax. (b) Extravasation from overlap zone indicative of type IIIa endoleak. (c) Intraprocedural fluoroscopy demonstrating balloon moulding in overlap zone. (d) Post-relining fluoroscopy shows no residual endoleak. (e) Re-lined stent graft extending just proximal to coeliac axis. (f) Three-week follow-up CT angiography shows intact thoracic endovascular aneurysm repair with resolving haemothorax with mixed-density blood products.

Given the hostile groins, access was obtained via left superficial femoral artery cut-down in a joint vascular surgery and interventional radiology procedure. Over a Lunderquist extra-stiff guidewire (Cook Medical) advanced to the ascending aorta, two Valiant Captivia thoracic stent-grafts (Medtronic) were deployed within the existing endograft across the rupture site (Supplementary Table S1). Balloon moulding of the overlap zone with a Reliant balloon (Medtronic) was undertaken and initial angiography showed no definite residual extravasation, but the patient remained profoundly haemodynamically unstable with unrecordable blood pressure and difficult ventilation.

Only at this point, with the defect covered, was a surgical chest drain inserted: the tense haemothorax was compromising ventilation and venous return, and the drain would quantify ongoing blood loss. The loss of any tamponade effect was accepted because the defect was covered and wire and sheath access were maintained, allowing immediate extension of the construct if bleeding continued. The drain yielded ~4 L; brisk recurrent drainage during groin closure prompted repeat angiography, which showed no discrete new leak. Repetitive aggressive balloon moulding at the graft overlap zone slowed and ultimately arrested the bleeding (Fig. 1c and d). Because the distal edge of the construct lay within a mildly ectatic segment, a third Valiant Captivia component was deployed to just above the coeliac axis to lengthen the distal landing zone and reinforce apposition (Fig. 1e). Final angiography demonstrated no residual endoleak with preserved visceral perfusion and maintained inflow to the prior aortobifemoral graft.

Post-procedure CT confirmed a continuous thoracic stent-graft construct without recurrent endoleak, but with a large residual left haemothorax, pelvic haematoma and multifocal thrombus/dissection in the left iliac-femoral axis. Follow-up CT 3 weeks later showed resolving haemothorax and no recurrent endoleak (Fig. 1f), and he was discharged home after prolonged critical care, with ongoing surveillance arranged at his local referring hospital, where further imaging was not available for this report.

### Case 2 – Delayed type IIIa endoleak progressing to thoracic rupture despite conservative surveillance

A 79-year-old man with chronic obstructive pulmonary disease and rheumatoid arthritis had a vascular history of open visceral debranching of the coeliac and superior mesenteric arteries, followed in 2008 by TEVAR with a modular thoracic stent-graft for a type B thoracoabdominal dissection (Fig. 2a); the make and model of the index device could not be retrieved from the archived records.

**Figure 2:**
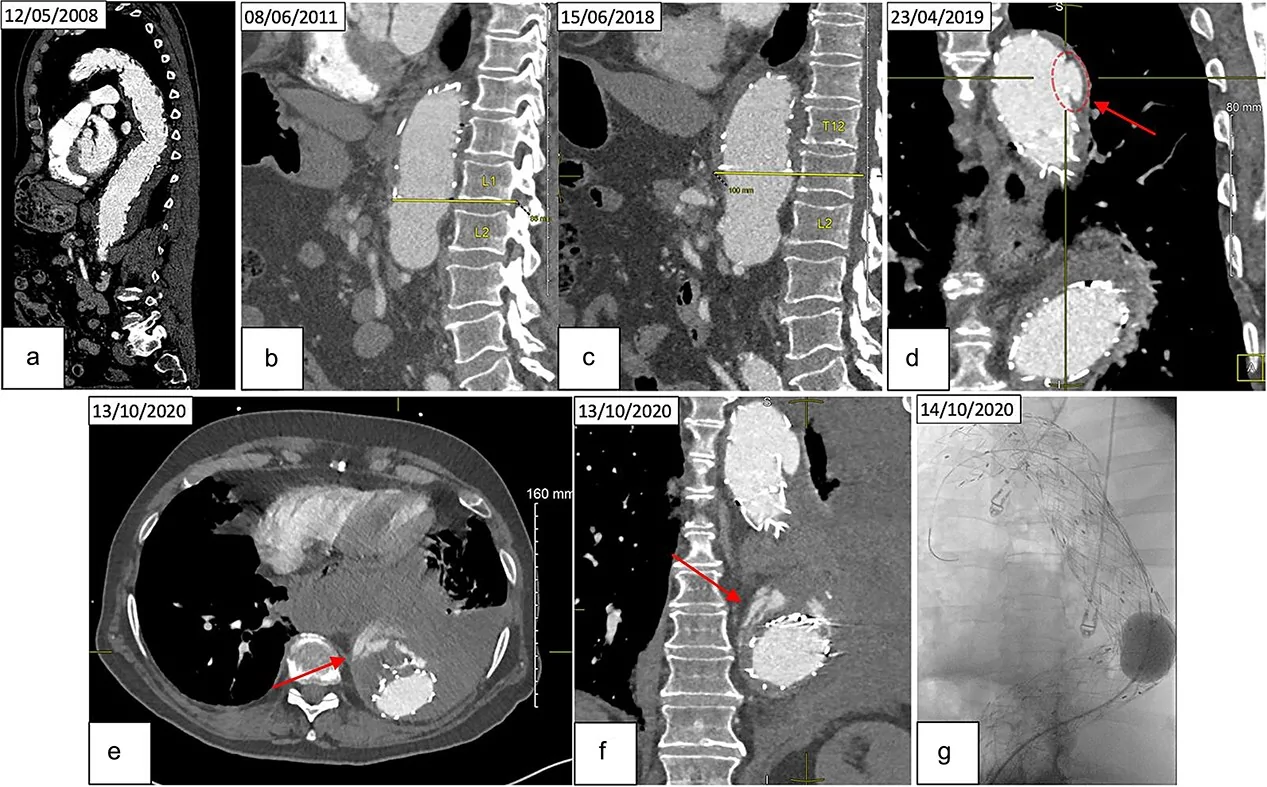
*Case 2: Evolution of delayed thoracic endovascular aneurysm repair failure from distal type Ib to proximal type IIIa endoleak, and subsequent acute thoracic rupture (panels a–g).* (a) Sagittal CT of post-thoracic endovascular aneurysm repair of type B thoracoabdominal aortic dissection. (b and c) Surveillance CT comparison from 08/06/2011 and 15/06/2018 demonstrating proximal migration of distal graft developing a type Ib endoleak and aneurysmal enlargement. (d) Sagittal CT from 23/04/2019 shows proximal type IIIa endoleak at thoracic overlap zone. (e and f) Axial and coronal CT on 13/10/2020 demonstrating acute rupture with left haemothorax and contrast extravasation from distal descending thoracic aorta. (g) Intraprocedural fluoroscopy of balloon moulding at overlap zone.

After a subsequent period without aortic imaging, CT in 2018 investigating suspected pancreatitis incidentally demonstrated proximal migration of the distal TEVAR component with new distal aneurysmal dilatation, consistent with a distal type Ib endoleak (Fig. 2b and c). The endoleak was abolished by distal extension of the TEVAR to the level of the right renal artery with a straight Valiant closed-web component secured with eight Heli-FX EndoAnchors (Medtronic).

Subsequent surveillance CT identified a new type IIIa endoleak at a thoracic overlap zone distal to the left subclavian artery, but no increase in overall aneurysm sac diameter (Fig. 2d). In view of the absence of sac growth and his comorbidities, principally cardiomyopathy with severe left ventricular impairment and moderate aortic stenosis, the multidisciplinary team (MDT) judged the risk of elective relining to outweigh the perceived risk of rupture from a non-expanding leak and elected conservative management with continued imaging surveillance. Whether emergency repair would be offered in the event of rupture was not explicitly considered or discussed with the patient at that time.

Two years later, aged 81, he presented acutely with severe chest and abdominal pain and with a systolic blood pressure ~60 mmHg. CT showed the extended TEVAR intact but active contrast extravasation from the distal descending thoracic aorta, anatomically separate from the unchanged proximal type IIIa endoleak, with massive left haemothorax and haemomediastinum (Fig. 2e and f).

Although judged unsuitable for elective relining, he had remained independent at home with heart failure symptoms controlled on medical therapy, he reached the tertiary centre alive, an endovascular option was technically feasible, and without intervention death was certain. With no previously agreed ceiling of treatment, the on-call team judged that the balance of risk now favoured emergency relining. Through a scarred left common femoral cut-down, three overlapping tapered Valiant Captivia components (Medtronic) bridged the areas of component separation and extended the repair distally (Fig. 2g). Completion angiography showed no residual endoleak, but after an intra-operative cardiac arrest and massive transfusion he remained in profound haemorrhagic shock and died 3 h later.

### Case 3 – Type IIIb arch endoleak salvaged via transoesophageal-guided left ventricular wire access

A 75-year-old man with hypertension, immune thrombocytopenic purpura and compensated cirrhosis was found to have a new type IIIb endoleak on surveillance CT following a complex staged aortic repair.

In 2019, a CT skeletal survey found an incidental chronic type B dissection with aneurysmal degeneration of the descending thoracic aorta, extending to the left common iliac artery with the coeliac and left renal arteries arising predominantly from the false lumen. A staged strategy was adopted: ascending aorta and arch replacement with a frozen elephant trunk (FET) and left subclavian bypass in July 2020; three Valiant Navion stent-grafts (Medtronic) from the FET to above the coeliac trunk in October 2020; and, after re-expansion of the distal thoracic sac to 78 mm, a custom four-fenestration Zenith thoracoabdominal graft (Cook Medical) with BeGraft (Bentley) and Advanta V12 (Getinge) bridging stents in 2022, landing proximally within the Navion segment. The short FET segment was within a highly angulated ‘gothic’ arch (Fig. 3a), which would later prove to be a key technical obstacle.

**Figure 3:**
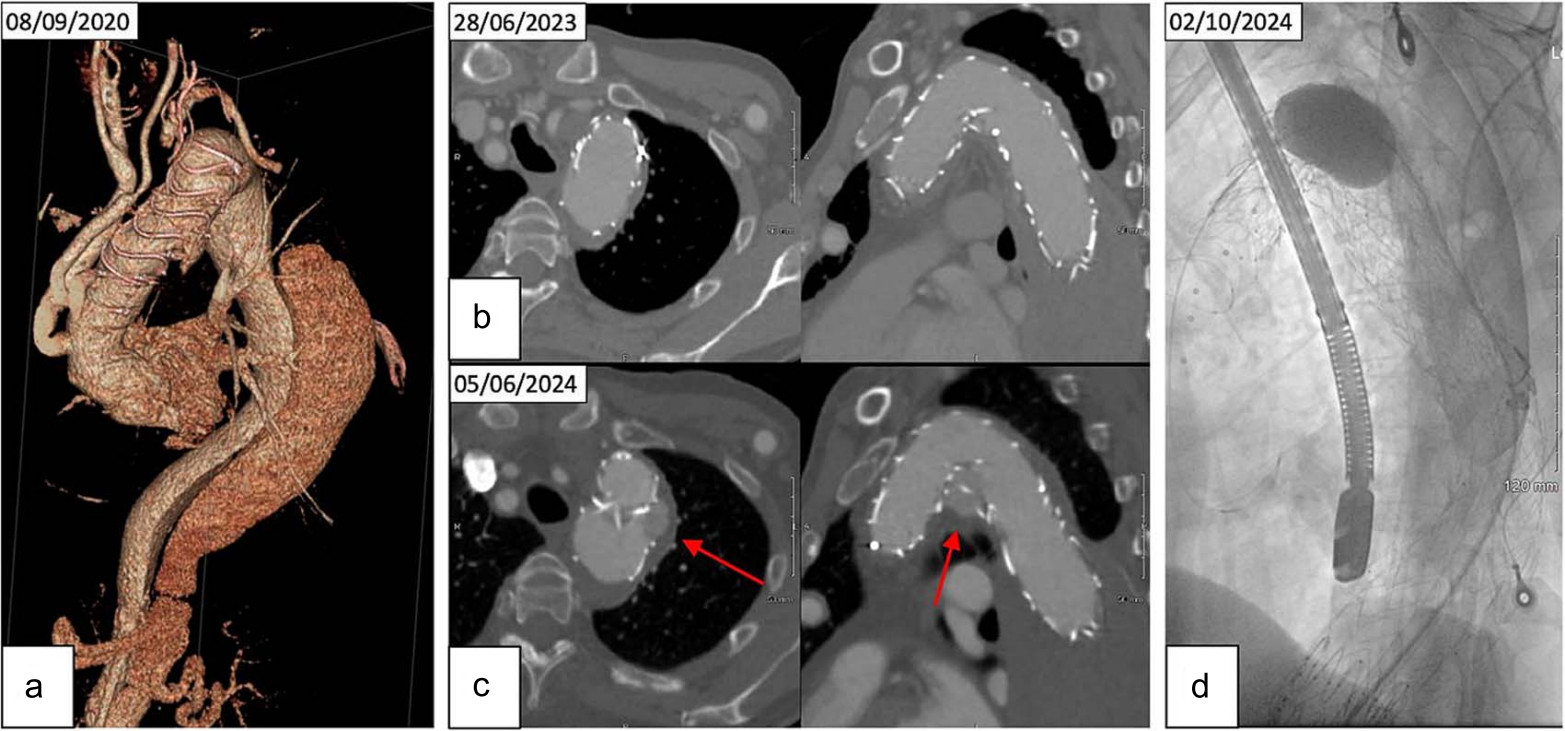
*Case 3: Type IIIb arch endoleak after staged repair and successful relining through a hostile aortic arch (panels a–d).* (a) Three-dimensional CT reconstruction showing the frozen elephant trunk and a markedly angulated (‘gothic’) aortic arch, with chronic type B dissection extending into the descending thoracic aorta. (b and c) Surveillance CT comparison from 28/06/2023 and 05/06/2024 demonstrating the development of a new type IIIb endoleak at the arch apex, just distal to the frozen elephant trunk overlap. (d) Intraprocedural fluoroscopy demonstrating relining of the thoracic arch stent-graft from the frozen elephant trunk across the highly angulated aortic arch.

On surveillance CT in June 2024, a new arch endoleak was identified just distal to the FET at the overlap between Navion components (Fig. 3b and c). Contrast lay outside the stent-graft fabric at the arch apex with an increase in arch diameter from 43 mm to 51 mm, consistent with type IIIb fabric failure, a recognized late failure mode of this device [9].

A first relining attempt in June 2024 used a Valiant Captivia device (Medtronic), the unit’s standard thoracic stent-graft sized to the 37 mm Navion components, via bilateral femoral access with right common femoral cut-down. Despite a Lunderquist extra-stiff guidewire, a second buddy Lunderquist wire and a Reliant balloon inflated in the arch to deflect the existing stents, the device would not track around the severely angulated arch and the procedure was abandoned after 1.5 L blood loss. A second attempt in October 2024 succeeded by modifying both approach and device (Fig. 3d). It was planned jointly with cardiothoracic surgery, with transapical left ventricular access held in reserve as a bail-out for a through-and-through wire [10]. Via a left groin cut-down, a double-curve Lunderquist wire was advanced across the aortic valve into the left ventricle under transoesophageal echocardiographic guidance, straightening the arch and providing a stable coaxial rail for a 24 F sheath. A GORE TAG conformable stent-graft with Active Control (W. L. Gore & Associates) was chosen for its lower profile, greater conformability and staged deployment permitting angulation control; it tracked around the arch apex into the FET and was deployed across the leak. Given the risk of further fabric failure, the whole Navion segment was relined with a second GORE TAG and a distal Valiant Captivia component overlapping into the fenestrated graft. Rapid ventricular pacing was not required. Completion angiography confirmed resolution of the type IIIb arch endoleak.

Six-week CT confirmed complete resolution of the endoleak and a stable sac, and CT at 5 and 12 months showed no new endoleak, sac expansion, dissection, rupture or device-related complication.

## Discussion

These three cases illustrate both the variability and severity of late type III endoleak after complex TEVAR. Three key themes emerge: the clinically catastrophic nature of type III endoleaks and the dilemma they pose in patients unfit for elective repair, the technical challenges of relining in hostile anatomy, and the benefits of consistent surveillance.

Firstly, type IIIa and IIIb endoleaks can have a clinically silent presentation, but can rapidly escalate into a vascular emergency. In Case 1, a small gap in the overlap between TEVAR graft modules precipitated massive haemothorax and near-fatal rupture. This mirrors findings from other studies where type III leaks were amongst the leading causes of late rupture after EVAR/TEVAR [11, 12]. Urgent relining was required to arrest the endoleak.

Case 2 raises a harder question. SVS and ESVS guidance for abdominal and thoracic repair regards any type III endoleak as failure of the repair that mandates treatment regardless of sac size [5–8]. We do not dispute this; the rupture in Case 2 supports it. The MDT decision to defer elective relining was nonetheless reasonable for a patient whose cardiac status made elective procedural risk appear prohibitive. What the case demonstrates is that such a decision is, in effect, a decision to palliate: the untreated leak carries a substantial, unpredictable rupture risk, and rupture may occur, as here, at a site separate from the known leak. When an MDT elects not to treat a type III endoleak on grounds of fitness, we suggest this be recognized explicitly as a palliative pathway, shared with the patient and family, and accompanied by a documented plan stating whether emergency repair would be offered in the event of rupture or care would be symptom-focused. In Case 2 no such plan existed, so when he presented in extremis the default was emergency intervention, carrying far higher mortality than the declined elective procedure. Early palliative care involvement and a documented escalation plan may serve such patients better than open-ended surveillance.

Secondly, a patient’s anatomy can challenge how endoleaks are managed, as exemplified in Case 3 through device failure at the apex of a highly angulated aortic arch [9]. In all three cases, relining required a stable proximal landing zone. The arch anatomy of Case 3 defeated standard transfemoral relining, necessitating left ventricular through-and-through wire support and a low-profile, highly conformable graft; a comparable strategy employing transapical access has been described previously [10].

Thirdly, these cases also highlight the importance of consistent surveillance, especially after complex repair, even when the sac is not expanding [13]. Case 1 also shows the vulnerability of patients whose follow-up is fragmented across centres. In Case 3, despite total aortic fixation, surveillance still discovered the type IIIb endoleak, showing that device failure remains possible, although rare. This is in line with current guidance, emphasizing the need for lifelong surveillance after FET and fenestrated/branched repair, with closer intervals in patients with high-risk devices or arch angulation [8].

Accurate imaging is central to detecting and classifying type III endoleaks, particularly subtle type IIIb fabric failures. Multiphasic CT angiography with careful assessment of overlap zones and interval sac change was sufficient here; dynamic, time-resolved CT angiography is a valuable adjunct for equivocal studies.

## Take home messages

- Late type III endoleaks after TEVAR may be radiologically subtle but are rapidly life-threatening, and a stable aneurysm sac alone should not defer elective re-intervention; the authors believe that these principles are equally applicable to EVAR.
- Conservative management of a type III endoleak on grounds of fitness is a palliative decision that should be shared with the patient and accompanied by a documented plan for rupture, which may occur at an anatomically separate site; lifelong surveillance remains essential.
- Successful relining of type IIIb endoleaks in highly angulated aortic arches may require conformable stent-grafts and left-ventricular through-and-through wire support, and these strategies should be anticipated and planned before intervention.

## Supplementary Material

JSCR_-_2026_-_1448_Supplementary_Table_S1_rjag887

## References

[1] Tohme S, Newman J, Yu PJ. Endovascular repair of thoracoabdominal aortic aneurysm: a brief review. Int J Angiol 2023;33:222–8. 10.1055/s-0043-1771343 PMC11534465 39502348

[2] Stoecker JB, Glaser JD. Review of type III endoleaks. Semin Interv Radiol 2020;37:371–6. 10.1055/s-0040-1715874 PMC7540642 33041482

[3] Blakeslee-Carter J, Beck AW, Spangler EL. Type 3 endoleaks in complex endovascular abdominal aortic aneurysm repair within the vascular quality initiative. J Vasc Surg 2022;75:1172–80. 10.1016/j.jvs.2021.10.038 PMC8940611 34740805

[4] Maleux G, Poorteman L, Laenen A et al. Incidence, etiology, and management of type III endoleak after endovascular aortic repair. J Vasc Surg 2017;66:1056–64. 10.1016/j.jvs.2017.01.056 28434700

[5] Chaikof EL, Dalman RL, Eskandari MK et al. The Society for Vascular Surgery practice guidelines on the care of patients with an abdominal aortic aneurysm. J Vasc Surg 2018;67:2–77.e2. 10.1016/j.jvs.2017.10.044 29268916

[6] Wanhainen A, Verzini F, Van Herzeele I et al. European Society for Vascular Surgery (ESVS) 2019 clinical practice guidelines on the management of abdominal aorto-iliac artery aneurysms. Eur J Vasc Endovasc Surg 2019;57:8–93. 10.1016/j.ejvs.2018.09.020 30528142

[7] Upchurch GR Jr, Escobar GA, Azizzadeh A et al. Society for Vascular Surgery clinical practice guidelines of thoracic endovascular aortic repair for descending thoracic aortic aneurysms. J Vasc Surg 2021;73:55S–83S. 10.1016/j.jvs.2020.05.076 32628988

[8] Wanhainen A, Gombert A, Antoniou GA et al. European Society for Vascular Surgery (ESVS) 2026 clinical practice guidelines on the management of descending thoracic and thoraco-abdominal aortic diseases. Eur J Vasc Endovasc Surg 2026;71:172–270. 10.1016/j.ejvs.2025.12.050 41448425

[9] Verzini F, Cieri E, Kahlberg A et al. A preliminary analysis of late structural failures of the Navion stent graft in the treatment of descending thoracic aortic aneurysms. J Vasc Surg 2021;74:1125–1134.e2. 10.1016/j.jvs.2021.04.018 33892122

[10] Martínez López I, Hernández Mateo MM, Maroto Castellanos LC et al. Thoracic stent grafting through transapical approach for type III Endoleak due to prosthetic disconnection. Ann Vasc Surg 2018;51:328.e1–5. 10.1016/j.avsg.2018.03.026 29778614

[11] Koudounas G, Bontinis V, Giannopoulos S et al. Rupture after previous endovascular abdominal aortic aneurysm repair: a meta-analysis and meta-regression analysis of factors influencing perioperative mortality. J Vasc Surg 2025;82:1097–110. 10.1016/j.jvs.2025.02.048 40120725

[12] Antoniou GA, Georgiadis GS, Antoniou SA et al. Late rupture of abdominal aortic aneurysm after previous endovascular repair: a systematic review and meta-analysis. J Endovasc Ther 2015;22:734–44. 10.1177/1526602815601405 26286073

[13] Sidloff DA, Stather PW, Choke E et al. Type II endoleak after endovascular aneurysm repair. Br J Surg 2013;100:1262–70. 10.1002/bjs.9181 23939840

