# Supplementary material for: Type III endoleaks following endovascular aortic repair: a case series
Source: J Surg Case Rep. 2026 Oct 4;2026(10):rjag887. doi: 10.1093/jscr/rjag887 (PMC13633880; doi:10.1093/jscr/rjag887)
Supplement: Supplementary file 1 [file jscr_-_2026_-_1448_supplementary_table_s1_rjag887.docx]

**Type III Endoleaks Following Endovascular Aortic Repair: Three Case Reports**

**Supplementary Table S1: Devices used**

Devices, guidewires and adjuncts used at each stage of treatment for the three cases, listed by case and procedure. Where the index thoracic stent-graft was implanted at another centre or before the current electronic record, and the make and model could not be retrieved, this is stated.

| **Case** | **Procedure** | **Device (model and size)** | **Manufacturer** |
| --- | --- | --- | --- |
| Case 1 | Index TEVAR (other centre, late 2023) | Modular thoracic stent-graft; make and model not available in transferred records | – |
| Case 1 | Emergency relining (April 2024) | Valiant Captivia thoracic stent-graft: FreeFlo 38 × 38 × 200 mm and Closed Web 38 × 38 × 150 mm across the defect; FreeFlo 38 × 38 × 200 mm distal extension | Medtronic, Minneapolis, MN, USA |
| Case 1 | Emergency relining (April 2024) | Lunderquist extra-stiff wire guide | Cook Medical, Bloomington, IN, USA |
| Case 1 | Emergency relining (April 2024) | Reliant stent-graft balloon catheter | Medtronic |
| Case 2 | Index TEVAR (2008) | Modular thoracic stent-graft; make and model not retrievable from archived records | – |
| Case 2 | Distal extension for type Ib endoleak (2018) | Valiant Closed Web thoracic stent-graft, straight 44 × 150 mm; Heli-FX EndoAnchor system (eight anchors) | Medtronic |
| Case 2 | Emergency relining for rupture (2020) | Valiant Captivia tapered thoracic stent-grafts × 3: 46–42 × 150 mm, 44–40 × 150 mm, 46–42 × 150 mm | Medtronic |
| Case 3 | Stage 1: ascending aorta and arch replacement (July 2020) | Frozen elephant trunk hybrid prosthesis with extra-anatomical left subclavian bypass | – |
| Case 3 | Stage 2: TEVAR (October 2020) | Valiant Navion thoracic stent-graft (VNMC) × 3: 37 × 31 × 207 mm, 37 × 31 × 207 mm, 37 × 37 × 182 mm | Medtronic |
| Case 3 | Stage 3: fenestrated thoracoabdominal repair (April and June 2022) | Custom four-fenestration Zenith thoracoabdominal graft (40–22 × 197 mm) with bifurcated distal body and Zenith Alpha Spiral-Z limbs | Cook Medical |
| Case 3 | Stage 3: bridging covered stents (2022) | BeGraft peripheral: right renal 6 × 28 mm, superior mesenteric 8 × 37 mm, coeliac 10 × 27 mm | Bentley InnoMed, Hechingen, Germany |
| Case 3 | Stage 3: bridging covered stents (2022) | Advanta V12: left renal 7 × 38 mm | Atrium Medical/Getinge, Hudson, NH, USA |
| Case 3 | First relining attempt (June 2024) | Valiant Captivia thoracic stent-graft 38 × 38 × 200 mm (could not be tracked; not deployed); Lunderquist extra-stiff wire guides × 2 (buddy-wire technique); Reliant balloon | Medtronic; Cook Medical |
| Case 3 | Definitive relining (October 2024) | Double-curve Lunderquist wire guide placed across the aortic valve for left ventricular through-and-through support; 24 F, 65 cm introducer sheath | Cook Medical |
| Case 3 | Definitive relining (October 2024) | GORE TAG Conformable Thoracic Stent Graft with ACTIVE CONTROL System × 2: 37 × 37 × 200 mm (22 F delivery profile) | W. L. Gore & Associates, Flagstaff, AZ, USA |
| Case 3 | Definitive relining (October 2024) | Valiant Captivia Closed Web thoracic stent-graft 40 × 40 × 150 mm (distal overlap into fenestrated graft) | Medtronic |

*TEVAR, thoracic endovascular aortic repair; F, French (delivery system size).*
